# Epidemiology and Risk Factors for Diarrheagenic *Escherichia coli* Carriage among Children in Northern Ibadan, Nigeria

**DOI:** 10.4269/ajtmh.22-0618

**Published:** 2023-10-30

**Authors:** Olabisi C. Akinlabi, El-shama Q. Nwoko, Rotimi A. Dada, Stella Ekpo, Adeola Omotuyi, Chukwuemeka C. Nwimo, Akinlolu Adepoju, Oluwafemi Popoola, Gordon Dougan, Nicholas R. Thomson, Iruka N. Okeke

**Affiliations:** ^1^ Department of Pharmaceutical Microbiology, Faculty of Pharmacy, University of Ibadan, Oyo, Nigeria;; ^2^ Medical Laboratory Science Program, College of Health Sciences, Bowen University, Iwo, Nigeria;; ^3^ Department of Pharmaceutical Microbiology, Faculty of Pharmacy, Ahmadu Bello University, Zaria, Nigeria;; ^4^ Department of Clinical Medicine, College of Medicine, University of Ibadan, Oyo, Nigeria;; ^5^ Department of Pediatrics, College of Medicine, University of Ibadan, Oyo, Nigeria;; ^6^ Department of Community Medicine, Faculty of Public Health, College of Medicine, University of Ibadan, Oyo, Nigeria;; ^7^ Wellcome Sanger Institute, Saffron Walden, United Kingdom;; ^8^ Department of Medicine, University of Cambridge, United Kingdom

## Abstract

Diarrhea is a leading cause of childhood morbidity in Africa, but few studies, focus on bacterial diarrheal etiology including multicountry studies that typically excluded Nigeria. We collected stool specimens from 477 children under 5 years of age, 120 with diarrhea, who were enrolled in our prospective case-control study between November 2015 and August 2019. All were attending primary health clinics on the northern outskirts of Ibadan. Up to 10 *Escherichia coli* isolates were obtained per specimen, and at least three of them were sequenced using Illumina whole-genome sequence technology. Genomes were assembled using SPAdes and evaluated for quality using QUAST. VirulenceFinder was used to identify virulence genes. The microbiological quality of water from 14 wells within the study area was assessed using total and coliform counts. Diarrheagenic *E. coli* (DEC) were isolated from 79 (65.8%) cases and 217 (60.8%) control children. A number of hybrid DEC pathotypes, *Salmonella* spp., *Yersinia* spp., and all DEC pathotypes except Shiga toxin-producing *E. coli* were detected, but no pathogen showed association with disease (*P* > 0.05). Enterotoxigenic *E. coli* were more commonly recovered from children without diarrhea aged below 6 months but exclusively detected in children with diarrhea aged over 9 months. Temporally linked, genetically similar enteroaggregative *E. coli* were isolated from children in different households in eight instances. No well water sample drawn in the study was potable. Children in northern Ibadan were commonly colonized with DEC. Access to water, proper sanitation, and vaccination against the prevailing pathogens may be critical for protecting children from the less overt consequences of enteric pathogen carriage.

## INTRODUCTION

Diarrhea has been reported to kill about 480,000 children each year worldwide. It accounted for 8% of all deaths in children less than 5 years of age in 2017,^1^ which translates to about 1,300 daily pediatric deaths, mostly in low- and middle-income countries. Nigeria has an untenably high number of deaths in children under 5 years of age annually,^2^ and in the year 2015, 15% of this mortality was attributed to diarrhea.^3^ Global diarrhea mortality declined by 61% between 1990 and 2020^2^ owing to improvements in preventive and treatment interventions. However, these improvements have less impact on morbidity, including in Nigeria.^4^^,^^5^

Understanding diarrhea etiology can help to discern and interrupt transmission pathways, overcome pathogen-specific risk factors, and provide valuable information to support vaccine and therapeutics development. There are numerous diarrhea pathogens, including enterovirulent bacteria, such as *Salmonella* and diarrheagenic *Escherichia coli* (DEC). Diarrheagenic *E. coli* isolates are classified based on virulence gene carriage and/or epithelial cell adherence pattern into different pathotypes, including *Shigella* and enteroinvasive *E. coli* (EIEC), Shiga toxin-producing *E. coli* (STEC), enterotoxigenic *E*. *coli* (ETEC), enterohemorrhagic *E. coli* (EHEC), enteropathogenic *E. coli* (EPEC), and enteroaggregative *E. coli* (EAEC).^6^ The epidemiology of the different DEC pathotypes varies geographically, and few studies have attempted to identify risk factors for DEC in childhood diarrhea.^7^^–^^13^

Diarrheagenic *E. coli* are neglected diarrheal pathogens, in part because they are challenging to differentiate from commensal *E. coli*. There have been only a handful of reports from Nigeria on the role of DEC in diarrhea. They range from the earliest studies of Agbonlahor and Odugbemi,^14^ Antai and Anozie,^15^ and Agbodaze et al.^16^ to work done in the last three decades by Ogunsanya et al.,^17^ Okeke et al.,^18^ Nweze,^19^ Onanuga et al.,^20^ Ifeanyi et al.,^21^ and Odetoyin et al.^22^ Nigeria (including Ibadan) is reported to have a high burden of feco-orally transmitted disease.^23^ These studies focused on only a subset of DEC pathotypes, and none was conducted in or around Ibadan.^24^ Previous studies have also not used methods sensitive enough to reliably and comprehensively identify DEC pathotypes. The HEp-2 cell adherence assay, the gold standard for identifying EPEC, EAEC, and another DEC category, diffusely-adeherent *E. coli* (DAEC) (but unable to identify other categories), is rarely performed in Nigeria; there is only one instance of use.^17^ The earliest studies used serotyping,^5^^,^^14^^,^^15^^,^^17^ which is only partially predictive for a few pathogenic subtypes (notably typical EPEC). The more recent studies used molecular targets; they identified each DEC pathotype by the use of only one or two gene targets.^18^^,^^22^^,^^25^^–^^30^ The use of a few identification targets is sufficient for some pathotypes, such as EPEC, ETEC, and EIEC, but not EAEC, which data suggest may be of significant epidemiological importance in this region^31^ and a neglected pathogen globally.^32^ This study aimed to associate the DEC pathotypes with diarrhea in children from Ibadan in southwestern Nigeria and to determine the social demographic risk factors associated with each DEC pathotype. Although we could not perform the HEp-2 adherence test, we aimed to expand sensitivity by screening for DEC targets through whole-genome sequencing (WGS).

## MATERIALS AND METHODS

### Study design.

This was a case-control study that enrolled children up to 5 years of age with and without diarrhea. Written informed consent was obtained from parents or guardians before samples were collected from the children. Case fecal samples were collected from children diagnosed with acute diarrhea by a health worker or community health officer. Controls were children attending the health center for vaccination deemed otherwise healthy by the health worker. Children were excluded from the study if they had been treated with antibiotics in the last month. A detailed questionnaire was used to obtain information on clinical history and physical examination findings. Mothers with no education or primary education were classified as having less than basic education, whereas those with secondary or tertiary education were considered to have basic education. Household water sources from borehole, well, bagged, and river sources were classified as unsanitary water, whereas boiled and bottled sources were classified as sanitary water.

### Sample sites and size.

**Figure 1.**
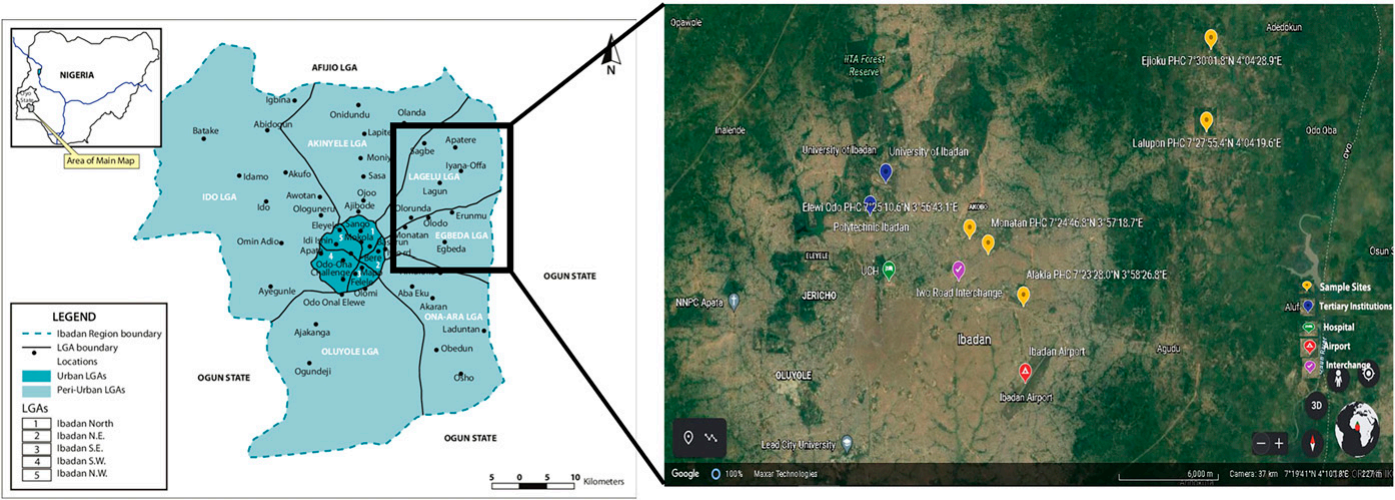
Study location. (**A**) Ibadan metropolis. (**B**) Location of the five participating primary health care centers. Ibadan metropolis comprises five central and six outlying local government areas (LGAs). The study LGAs, Lagelu and Egbeda, are in the northeastern suburbs.

The study was conducted on the northern outskirts of Ibadan, the state capital of Oyo State commonly known as the city of the red roofs due to its ancient buildings. The study enrollment points were five different primary health centers in Lagelu and Egbeda local government areas (LGAs), which lie immediately northeast of the metropolis (Figure 1). These LGAs are semi-urban and lack good access to water and sanitation.

### Sample processing, culture, and bacterial isolation.

Stool specimens were transported to the laboratory within 1 to 2 hours of collection. Samples were placed on eosin methylene blue agar (Oxoid), MacConkey agar plates (Oxoid), and selenite F broth and incubated at 37°C for 18 hours. Five representative non-lactose-fermenting colonies and five lactose-fermenting colonies were picked and subcultured to obtain pure cultures. Growth from the selenite F broth was further streaked on xylose lysine deoxycholate agar to obtain black-centered colonies with slightly red edges. The morphological appearances (color, shape, edge, and colony surface) of all isolates were noted, and isolates were stored by freezing at −80°C in 1:1 Luria broth-glycerol stock. Presumptive *E. coli* isolates were primarily identified biochemically using the Microbact 24E system (Oxoid), and *Salmonella* spp. were confirmed by *invA* polymerase chain reaction (PCR).^33^

### Occult blood testing.

Occult blood was detected using a qualitative immunoassay kit for hemoglobin (Cromatest) in accordance with the manufacturer’s instructions. Fresh stool samples were emulsified and placed on the sample section containing the particle coated with anti-hemoglobin antibody of the kit, and a drop of the buffer was added to it and then timed for 1 minute for the stool and buffer mix to migrate upward by capillary action. The presence of two lines (control and positive lines) indicates that the reaction is positive, and a single line (control line) indicates a negative result.

### DNA extraction, sequencing, and sequence analyses.

From each specimen, at least three primarily identified *E. coli* isolates were sequenced. The *E. coli* strains were selected based on their different Microbact 24E and morphological profiles. DNA was extracted using the Wizard genomic extraction kit (Promega) per the manufacturer’s protocol, and then they were library prepared with the NEBNext Ultra II FS DNA library kit and whole genome sequenced on the Illumina platform at the Wellcome Sanger Institute. Raw read quality control was carried out using FastQC (Babraham Bioinformatics, Babraham Institute, Cambridge, United Kingdom), and quality reports were aggregated using MultiQC.^34^ Reads were assembled using the SPAdes assembler, and assembly quality was determined using the Quality Assessment Tool for Genome Assemblies (QUAST)^35^ and CheckM.^36^ Reads were also assigned taxonomic identities using Kraken, and Bracken was used to determine species abundance of the taxonomic identities assigned by Kraken.^37^^,^^38^ The virulence genes were identified using the ARIBA VirulenceFinder database.^39^ Isolate genomes were deposited in the European Nucleotide Archive (ENA) under project ID PRJEB8667 (https://www.ebi.ac.uk/ena/browser/view/PRJEB8667).

### Identification of DEC pathotypes from whole-genome sequence.

The VirulencFinder output was used to classify *E. coli* into pathotypes. Sequenced *E. coli* isolates with any of the genes *aaiC, aar, aap, aatA (CVD432), aggA, aafA, agg3A, agg4A, agg5A, aggR, air, capU,* and *eilA* were classified as EAEC. *Escherichia coli* with either locus of enterocyte effacement (LEE) genes (including *eae*) and *bfp* or LEE genes without *bfp* or *stx* genes are classified as EPEC. *Escherichia coli* with either *sta* (heat-stable enterotoxins [ST] of ETEC) and/or *ltcA* (heat-labile enterotoxins of ETEC) with or without *lngA* were classified as ETEC. Sequenced *E. coli*/*Shigella* with either *ipaD, ipaH,* and/or *virF* were classified as EIEC/*Shigella.* Any strain with any *stx* gene was categorized as STEC, whereas those also harboring LEE genes were considered EHEC.

### Sentinel water quality analysis.

One liter of water was collected from 15 water sources (all wells) that were proximal to the health centers during the second year of the study. The water samples were collected aseptically into sterile bottles and transported to the laboratory on ice for analysis within an hour. The pH was measured using an HI 2210 pH meter (Hanna Instruments), total counts were computed after plating on tryptic soy agar (Oxoid), and coliform counts were performed on MacConkey agar plates (Oxoid). Isolates were identified the same way that the stool isolates were identified.

### Statistical analysis.

For data analysis, Epi Info version 7 software (Centers for Disease Control and Prevention, Atlanta, GA) and SPSS (Statistical Package for Social Science) version 20 software were used. Fisher’s exact and χ^2^ tests were used to examine the relationship between cases and controls and various pathotypes and genes, whereas bivariate analysis and logistic regression were used to assess the relevance of possible risk variables. *P* values < 0.05 were considered statistically significant, and Bonferroni corrections were made, where appropriate.^40^

## RESULTS

### High rates of recovery of diarrheagenic *E. coli* from cases and controls.

**Figure 2.**
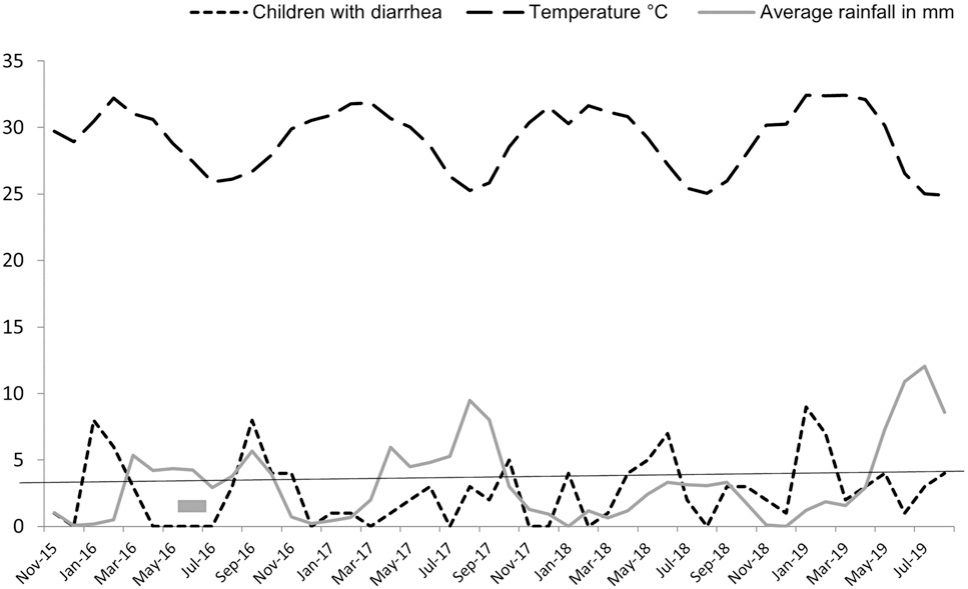
Monthly recruitment of children with diarrhea (dotted line) with the average rainfall (solid line) and median temperature (dashed line) recorded during the study, from 2016 to 2019.

Stool specimens from 120 consenting patients with diarrhea and 357 apparently healthy controls attending primary health care facilities in Egbeda and Lagelu local government areas of Ibadan, Nigeria, were processed between November 2016 and August 2019. Figure 2 shows how case enrollment of those less than 5 years of age was distributed over the year from November 2015 to August 2019. As Figure 2 indicates, there was a health system strike between June 7, 2016 and July 25, 2016, during which time patients could not be recruited. Figure 2 also shows temperature and rainfall distribution of Oyo State, over the sample collection period, revealing no association between rainfall, temperature, and diarrhea case recruitment during the study.

Diarrheagenic *E. coli* pathotypes were isolated from 323 (68.6%) of the stool specimens and include 281 EAEC (58.9%), 23 EPEC (4.8%), 19 ETEC (4.0%), and 1 EIEC (0.2%). There were no STEC and EHEC strains, as none of the sequenced *E. coli* carried the *stx1* and/or *stx2* genes (encoding Shiga toxins 1 and 2). Enteroaggregative *E. coli* was the most frequently recovered pathotype. Enterotoxigenic *E*. *coli* strains were recovered from 8 cases and 11 controls (*P =* 0.0823), and ETEC strains bearing the ST-encoding gene (*sta*) were isolated only from patients with diarrhea. However, no pathotype was significantly associated with children presenting with or without diarrhea. A total of 32 isolates met the definition of more than one pathotype; thus, we categorized these as pathotype hybrids. Except for one ETEC-EPEC hybrid, the hybrids all carried EAEC genes, with EAEC-ETEC and EAEC-EPEC being the most common (Table 1). No hybrid pathotype was associated with diarrhea.

**Table 1:** DEC pathotypes isolated from samples from children with and without diarrhea (control)

Pathotypes	Children with diarrhea (%) (*n* = 120)	Healthy children (%) (*n* = 357)	Total (*N* = 477)	*P* value	Odds ratio	95% CI (lower–upper)
EAEC	72 (60)	209 (58.5)	281	0.7790	1.0622	0.6968–1.6192
EAEC-EIEC (*capU*-*ipaH*)	0	1	1	1.0000		
EAEC-ETEC (*capU*/*eatA*/*air*/*eilA*/*aap*/*ltcA*)	5	7	12	0.1876		
EAEC-EPEC (*aar*/*agg4A*/*air*/*eilA*/*aggR*/*capU*/*aatA*-*eae*)	4	6	10	0.2794		
EPEC	7 (5.8)	16 (4.5)	23	0.5499	1.3202	0.5297–3.2908
*eae*	7 (5.8)	16 (4.5)	23	0.5499		
*bfp+eae* (typical EPEC)	1 (0.8)	2 (0.6)	3	1		
ETEC	8 (6.7)	11 (3.1)	19	0.0823	2.2468	0.8818–5.7248
*sta*	2 (1.7)	0 (0)	2	0.0629		
*ltcA*	7 (5.8)	11 (3.1)	18	0.1711		
*sta+ltcA*	1 (0.8)	0 (0)	1	0.2516		
*ltcA+lngA*	1 (0.8)	0 (0)	1	0.2516		
ETEC-EPEC	1	0	1	0.2526		
EIEC/*Shigella*	0 (0)	1 (0.3)	1	0.3368	0.0000	0-∞
*ipaH*	0 (0)	1 (0.3)	1			
*ipaD*	0 (0)	1 (0.3)	1			

DEC = diarrheagenic *Escherichia coli*; EAEC = enteroaggregative *Escherichia coli*; EIEC = enteroinvasive *Escherichia coli*; ETEC = enterotoxigenic *Escherichia coli*; EPEC = enteropathogenic *Escherichia coli*.

**Figure 3.**
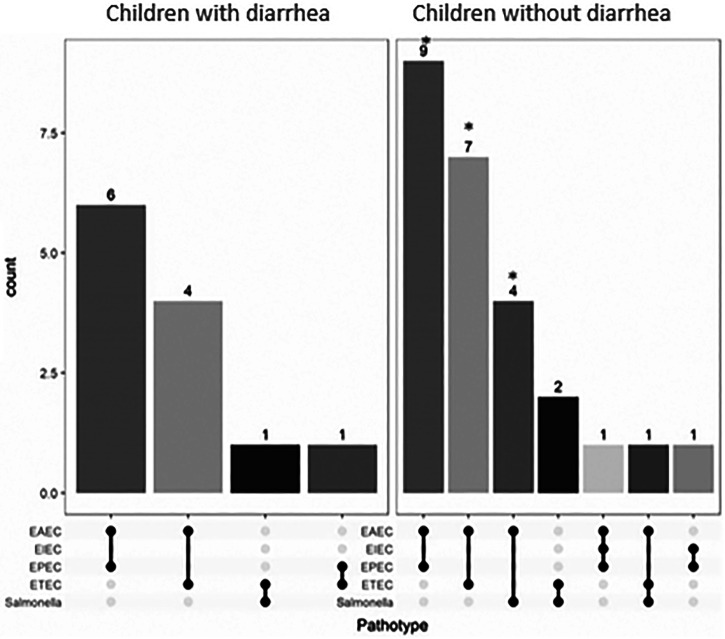
Diarrheic *Escherichia coli* pathotype or *Salmonella enterica* coinfection rates. An asterisk indicates that the observed frequency of the coinfection exceeded expectation based on the recovery of single infections among healthy children but not children with diarrhea (*P* ≤ 0.0025).

The high infection/carriage rates reflect that more than one pathogen was recovered from many individuals in the study. We computed, based on observed recovery rates for each pathogen, the expected frequency of recovered combinations of DEC pathogens or *Salmonella*. Figure 3 shows that most coinfections were detected at the expected level, but the observed coinfection levels of EAEC-EPEC, EAEC-ETEC, and EAEC-*Salmonella* exceeded expectation among children without diarrhea but not children with diarrhea (*P* < 0.0025). Indeed, *Salmonella* was always recovered with a DEC isolate from controls, and in one of three cases, this isolate was ETEC.

Bacterial species isolated included *E. coli* (commensal and DEC), *Klebsiella* spp., *Yersinia* spp., and *Salmonella* spp. (Table 2). As with the DEC isolates, *Salmonella* spp. and *Yersinia* spp. were not associated with diarrhea. However, they were isolated proportionately more in cases than controls and were uncommonly recovered. There was a greater diversity of non-enteric pathogen genera in controls than in cases, and *Klebsiella*, an abundant and commonly carried commensal, was significantly less frequently detected in cases than in controls.

**Table 2:** *Enterobacterales* and related genera isolated at least once from children enrolled in the study

Species and genera*	Children with diarrhea (*n* = 120)	Healthy children (*n* = 357)	Total	*P* value	Odds ratio	95% CI (lower–upper)
*Escherichia coli*	106	307	413			
*Escherichia hermanii*	2	10	12			
*Escherichia fergusonii*	1	2	3			
*Escherichia vulneris*	0	2	2			
*Shigella sonnei*	0	1	1			
**Genus *Escherichia***	**109**	**322**	**431**	**0.7189**	**1.1386**	**0.5613–2.3096**
*Acinetobacter baumannii*	1	3	4			
*Acinetobacter haemolyticus*	3	5	8			
*Acinetobacter lwoffii*	1	4	5			
**Genus *Acinetobacter***	**5**	**12**	**17**	**0.7759**	**1.2494**	**0.3375–3.9113**
*Citrobacter diversus*	2	5	7			
*Citrobacter braakii*	0	1	1			
*Citrobacter murliniae*	0	2	2			
*Citrobacter amalonaticus*	0	2	2			
*Citrobacter freundii*	0	6	6			
*Citrobacter youngae*	0	1	1			
**Genus *Citrobacter***	**2**	**17**	**19**	**0.1795**	**0.3390**	**0.0375–1.4649**
*Cronobacter sakazakii*	0	2	2			
*Edwardsiella tarda* biogroup 1	0	1	1			
*Enterobacter cloacae*	0	9	9			
*Enterobacter agglomerans*	1	5	6			
*Enterobacter aerogenes*	1	5	6			
*Enterobacter gergoviae*	1	9	10			
**Genus *Enterobacter***	**3**	**28**	**32**	**0.0516**	**0.3013**	**0.0577–1.0053**
*Ewingella americana*	1	0	1			
*Hafnia alvei*	2	12	14			
*Klebsiella oxytoca*	2	18	20			
*Klebsiella ozaenae*	1	8	9			
*Klebsiella pneumoniae*	2	25	27			
*Klebsiella terrigena*	0	2	2			
**Genus *Klebsiella***	**8**	**79**	**87**	**0.0001**	**0.2514**	**0.1176–0.5372**
*Kluyvera ascorbata*	1	1	2			
*Kluyvera cryocrescens*	1	0	1			
*Morganella morganii*	2	7	9			
*Proteus mirabilis*	0	7	7			
*Providencia rettgeri*	1	3	4			
*Providencia stuartii*	0	2	2			
**Genus *Providencia***	**1**	**5**	**6**	**1.0000**	**0.5916**	**0.0124–5.3682**
*Salmonella* Riverside	0	2	2			
*Salmonella* Elisabethville	1	0	1			
*Salmonella* Give	0	2	2			
*Salmonella* Agama	0	1	1			
*Salmonella* Stanleyville	1	0	1			
**Genus *Salmonella***	**3**	**6**	**9**	**0.5682**	**1.4986**	**0.2388–7.1492**
*Serratia liquefaciens*	0	9	9			
*Serratia marcescens*	3	12	15			
*Serratia odorifera*	0	2	2			
*Serratia rubidaea*	0	1	1			
**Genus *Serratia***	**3**	**24**	**27**	**0.1088**	**0.3564**	**0.0675–1.2063**
*Stenotrophomonas maltophilia*	0	1	1			
*Yersinia enterocolitica*	1	0	1			
*Yersinia ruckeri*	0	2	2			
**Genus *Yersinia***	**1**	**2**	**3**	**1.0000**	**1.4902**	**0.0251–28.8643**

* Individual species are listed and totals for each genus are indicated in bold text.

### Risk factors for DEC carriage.

**Figure 4.**
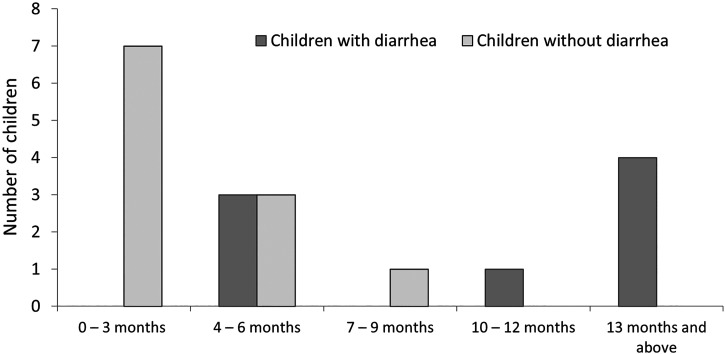
Age distribution of children from whom enterotoxigenic *Escherichia coli* (ETEC) was recovered.

Although ETEC was more commonly isolated from younger controls and older cases (Figure 4), age was significantly associated with ETEC infection among the cases (Supplemental Table 3). Mother’s education level was significantly associated with ETEC (*P* < 0.05) (Supplemental Table 3), while the logistic regression shows that children whose mothers do not have basic education were 4 times more likely to have ETEC than children whose mothers have basic education (*P* < 0.05) (Supplemental Table 5). Bivariate analysis of parameters describing the nutritional status of children and the household water sources of children from whom DEC pathotypes were recovered did not associate any variables with DEC recovery (Supplemental Tables 1–4).

### EAEC carriage clusters.

As Figure 2 shows, cases were unevenly reported from the participating primary health care centers. The overall median number of children with diarrhea recruited per month across the study was 3, but the range included months with up to nine enrollments. In months January to February 2016, September 2016, June 2018, and January to February 2019, the number of children with diarrhea recruited was above 2 standard deviations of the mean of 3. Within these months, EAEC isolation was overrepresented, and genetically identical EAEC isolates were recovered from two individuals within each of these clusters (Table 3). At least one of the strains in each cluster pair was isolated from a child with diarrhea. Thus, identical EAEC strains were recovered within the detected clusters, but we lack evidence to propose diarrhea outbreaks per se. All the clusters occurred in drier months (rainfall < 4 mm).

**Table 3:** List of EAEC clusters

EAEC strains	Number of individuals involved	Same or different household	*E. coli* sequence type	Study participants	Enrollment site (primary care clinic)	Month/year of isolation
LLH051B/LLH059E	2	Yes	501	Healthy	Lalupon	Jan 2016, Feb 2016
LWD015H/LLH054C	2	No	648	Diarrhea, healthy	Lalupon	Jan 2016, Feb 2016
MNH226F/MNH225J	2	Yes	69	Healthy	Monotan	Jun 2018, Jun 2018
MNH227C/MND75D	2	Yes	40	Diarrhea, healthy	Monotan	Jun 2018, Jul 2018
MNH224F/JKD76F	2	No	1808	Diarrhea, healthy	Monotan, Ejioku	Jun 2018, Jun 2018
LLH316ABCDE/LLH319C	2	Yes	8746	Healthy	Lalupon	Feb 2019, Mar 2019
LLH311A/LLH315C	2	Yes	10	Healthy	Lalupon	Jan 2019, Feb 2019
LLH315G/LLH311G	2	Yes	1722	Healthy	Lalupon	Jan 2019, Feb 2019

EAEC = enteroaggregative *Escherichia coli*; Feb = February; Jan = January; Jul = July; Jun = June; Mar = March; ST = sequence type.

### Total and coliform counts from sentinel water samples.

Total counts of the water samples collected from 15 wells in the study area approached or exceeded 10^3^ colony-forming units (CFU)/mL, and coliform counts ranged between 10^1^ and 10^4^ CFU/mL (Table 4). The presence of *E. coli*, indicating recent fecal contamination, was recorded in 10 of the 14 well samples. *Salmonella* were enriched for but were not cultured from any of the wells. The control water sample, UIH202, was collected from a local water bottling plant and had a high bacterial count but no coliforms or *E. coli* (Table 4).

**Table 4:** Total coliform and total bacterial counts from sentinel water samples

Sample	Total bacterial count (CFU/mL)	Total coliform count (CFU/mL)
UIH202	1.60 × 10^3^	None
LLH201	1.63 × 10^3^	6.90 × 10^2^
LWH203	2.18 × 10^3^	5.70 × 10^2^
LWH204	1.81 × 10^3^	3.40 × 10^2^
LWH205	6.10 × 10^3^	6.00 × 10^1^
LWH206	2.20 × 10^3^	6.00 × 10^1^
LWH207	2.42 × 10^3^	4.90 × 10^2^
LWH208	2.37 × 10^3^	1.90 × 10^2^
LLH209	2.11 × 10^3^	3.60 × 10^2^
LLH2010	2.38 × 10^3^	1.30 × 10^2^
LWH2011	7.30 × 10^2^	4.00 × 10^2^
LWH2012	1.87 × 10^3^	2.40 × 10^2^
LWH2013	7.60 × 10^3^	1.00 × 10^2^
LWH2014	4.80 × 10^3^	1.20 × 10^2^
UIH2015	4.70 × 10^3^	7.00 × 10^1^
LWH2016	2.30 × 10^3^	1.00 × 10^2^

CFU = colony-forming units.

## DISCUSSION

This study sought DEC pathotypes in specimens from 120 children with diarrhea and 357 children without diarrhea. Our study participants were recruited from primary care centers; therefore, the children presented with expectedly mild diarrhea. Diarrhea etiology studies often exclude children with the more common mild diarrhea episodes because they are unlikely to reach hospitals, where most diarrheal epidemiology studies are conducted.^7^^,^^13^^–^^17^^,^^25^^,^^41^ We isolated up to 10 colonies per specimen, ultimately recovering *E. coli* at least once from 106 (88.3%) children with diarrhea and 307 (86%) children without diarrhea. We additionally enriched for *Salmonella* spp. As is typical in studies of this nature, *E. coli* was the predominant *Enterobacterales* species recovered. The diversity of enteric Gram-negative bacteria was less in fecal specimens from children with diarrhea than in those from children without diarrhea. Common enteric commensals such as *Klebsiella* spp., *Enterobacter* spp., and *Citrobacter* spp. were recovered less commonly from cases than controls. Other unequivocal pathogens were recovered, including *Yersinia* (for which we did not enrich) and *Salmonella* (directly or after selenite broth enrichment). Although these genera are commonly associated with diarrhea in a range of studies, in this study they were proportionately more common in cases than controls but were uncommonly recovered and not associated with diarrhea (*P* > 0.05). This is reflective of the epidemiology we observed for DEC pathotypes and of the high levels of enteric pathogen carriage we documented.

*Salmonella* spp., *Yersinia* spp., EAEC, ETEC, EPEC, and EIEC were isolated in this study but showed no association with diarrhea.^17^^,^^20^^,^^42^^–^^44^ EHEC and STEC were not recovered and typically are not featured in diarrhea studies of children less than 5 years of age in Africa. Screening multiple isolates per individual and identifying *E. coli* subtypes based on WGS permitted the detection of 537 DEC isolates (including atypical strains), a much higher detection rate than in comparable studies, and may also account for our high detection rate in controls. Ultimately, at least one DEC isolate was recovered from 77 (64.2%) cases and 220 (61.6%) controls.

A hybrid pathotype contains virulence or other loci that would lead to its being classified as more than one DEC pathotype, sometimes conferring greater virulence. Hybrids have been associated with outbreaks,^45^^–^^47^ but outbreak investigations typically prompt more comprehensive isolate characterization and are therefore more likely to detect hybrids. In this study, 32 (2.6%) DEC isolates were hybrid DEC pathotypes. Enteroaggregative *E. coli*-EPEC hybrids, similar to an EAEC–atypical-EPEC hybrid with the *eae* and *aggR* genes reported from Brazil in 2021,^47^ were identified in 10 specimens (four from children with diarrhea and six from healthy children; not statistically significant). We additionally detected EAEC-ETEC in 16 specimens (five children with diarrhea and seven healthy children; not statistically significant), ETEC-EPEC in two specimens from children with diarrhea, and EAEC-EIEC in one specimen from a healthy child.

Enteroaggregative *E. coli*, which is increasingly sought in diarrheal epidemiology studies and reported in African studies,^7^^,^^17^^,^^21^^,^^25^^,^^31^^,^^48^^,^^49^ was the most commonly isolated DEC pathotype. Enteroaggregative *E. coli* has been associated with diarrhea in some epidemiological studies in Africa^7^^,^^17^^,^^31^^,^^41^ but not in others.^25^^,^^48^^,^^49^ High EAEC recovery from both children with diarrhea and children without diarrhea has been reported in Nigeria^17^^,^^18^^,^^21^ and in multiple locations in the MAL-ED study in Brazil, Bangladesh, Tanzania, India, Peru, South Africa, Pakistan, and Nepal.^50^ There is evidence that EAEC carriage may lead to nutrient malabsorption, in addition to or instead of diarrhea.^7^^,^^50^ Hence, the high carriage rates we observed are concerning despite being unassociated with frank diarrhea. Altogether, EAEC was isolated from 281 (58.9%) children in this study, representing 95% of 296 children from whom DEC were recovered. Enteroaggregative *E. coli* was recovered with another DEC pathotype from 23 specimens and, in the case of EPEC, more commonly than combined probabilities would predict (*P* = 0.0025). Chattaway et al.^51^ have also reported that certain other pathogen combinations are common with EAEC, and although their significance is unclear, these combinations warrant further investigation.

The ETEC detection rate in this study was high compared with that of previous studies in the region.^18^ Enterotoxigenic *E*. *coli* were recovered from 8 (6.7%) children with diarrhea and 11 (3.1%) healthy children (*P =* 0.0823). Enterotoxigenic *E*. *coli sta* gene, which encodes the heat-stable enterotoxin and is more commonly associated with diarrhea,^52^ was detected only in isolates from children with diarrhea, and one isolate harbored both the *ltcA* and *sta* genes. Enterotoxigenic *E*. *coli* was more common in younger children without diarrhea, aged 0 to 6 months, and older children with diarrhea, aged 13 months and above. The bivariate analysis confirmed age to be associated with ETEC infection, while logistic regression revealed that age groups below nine months are associated with ETEC infection. Weaned children may lose protection from secretory IgA present in breast milk, which has previously been reported to be protective for ETEC.^53^ Also, crawling and weaning may increase the exposure of older children to ETEC through the consequent ingestion of infected material from food and drink, and their surroundings may amplify their risk.^54^ However, our data strongly suggest that young children are exposed to ETEC but are simply not sickened by it. The poor quality of household water, which may be used for bathing babies, may be one of many risk factors. In comparison to ETEC, the much higher carriage rates for EAEC and the age-unspecific risk may in turn suggest that breast milk does not protect against EAEC. It is, however, important to highlight that exclusive breastfeeding was not found to be protective against diarrhea or carriage of any of the pathotypes in this study. However, nonexclusive breastfeeding was not queried.

Enteropathogenic *E. coli* are pathogens almost exclusively associated with childhood diarrhea. In the current study, EPEC was isolated from 7 (5.8%) diarrheal specimens and 16 (4.5%) specimens from healthy children. Two isolates from a healthy child and one isolate from a child with diarrhea were typical EPEC strains with both *eae* (with other LEE genes) and *bfpA* genes. Atypical EPEC strains carrying LEE genes including *eae* but lacking the *bfpA* gene were isolated from seven children with diarrhea and 16 healthy children. Enteropathogenic *E. coli* has been historically prominent in Nigeria,^14^^,^^16^ based on previous studies that used serotyping. However, more recent studies using DNA hybridization^17^ and PCR,^18^^–^^20^^,^^55^^,^^56^ like our current WGS study, have found this pathotype to be much less common, suggesting that earlier results may be due to methodological artefacts.

*Shigella* is reportedly a principal diarrheal agent in African countries and has consequently been prioritized for vaccine development.^57^^–^^61^ EIEC, which have similar virulence factors, may account for at least some of the global burden of *Shigella*.^17^^,^^41^^,^^62^ In the current study, only 1 (0.3%) *Shigella sonnei* isolate (from a control) and no EIEC were recovered. This study also included only four cases of bloody diarrhea, two reported by patient caregivers, two others detected by fecal occult blood testing, and none from which *Shigella* or EIEC were recovered. The relatively low recovery of *Shigella* in this study may be due to our focus on largely mild diarrhea, treated close to home at primary health care facilities. Other similarly focused studies have made comparable findings.^17^^,^^63^ Reports in the literature largely focus on hospital-managed diarrhea, which is more likely to be moderate to severe diarrhea, and might be why *Shigella* is associated with diarrhea in those studies.

Similar to other African study reports,^64^^–^^66^ a recent study in Cape Town, South Africa,^67^ identified water sources and storage as diarrhea risk factors. We observed that household and shared wells were the most common water sources used by residents in the primary health care center areas (Supplemental Table 3). When we sampled and analyzed water quality from 15 wells, we observed that all samples analyzed had high bacterial and coliform loads exceeding the 20 CFU/mL and < 1 CFU/mL potable water limits, respectively.^68^ *Escherichia coli*, the standard indicator of fecal contamination, was isolated from 11 (78.6%) water samples, suggesting that water and sanitation improvements should be primary priority interventions. Despite exclusive breastfeeding and the use of presumed potable drinking water (such as bottled or bagged water) for children, the very use of unpotable household water for bathing and washing places very young children at risk of diarrhea.

In conclusion, this study found worryingly high carriage rates for DEC and other bacterial pathogens with known diarrheal roles. Children in northern Ibadan were commonly colonized with EAEC and other bacterial pathogens, including ETEC, EPEC, *Shigella, Salmonella*, and *Yersinia*, as well as with DEC hybrids. Although we did not find associations with diarrhea despite these high carriage rates, pathogens were frequently recovered from children with diarrhea reporting to primary health care centers. Asymptomatic colonization is common and is more pronounced after weaning. Moreover, identical EAEC strains were frequently recovered from children residing in different households, suggesting the circulation of feco-orally transmitted pathogens in the study area. No well water sampled from a selection of households in the vicinity of the primary care centers was potable; thus, improving access to water and sanitation may be critical for protecting children from the less overt consequences of enteric pathogen carriage in northern Ibadan.

## Financial Disclosure

This work was supported by an African Research Leader’s Award (to I. N. O., G. D., and N. R. T.), which was jointly funded by the United Kingdom Medical Research Council (MRC) and the United Kingdom Department for International Development (DFID) under the MRC/DFID Concordat agreement and is also part of the EDCTP2 program supported by the European Union. I. N. O. is a Calestous Juma Fellow supported by the Bill and Melinda Gates Foundation (Grant no. INV-036234).

## Supplemental files

10.4269/ajtmh.22-0618 Supplemental Materials
