# Supplementary material for: Epidemiology and Risk Factors for Diarrheagenic Escherichia coli Carriage among Children in Northern Ibadan, Nigeria
Source: Am J Trop Med Hyg. 2023 Oct 30;109(6):1223–32. doi: 10.4269/ajtmh.22-0618 (PMC10793065; doi:10.4269/ajtmh.22-0618)
Supplement: Supplemental Materials [file tpmd220618.SD1.pdf]

Supplementary Table 1: Social Demographic Characteristics of Children with DEC pathotypes

Variable	Case			Control		
	All DEC		X^2 (p-value)	All DEC		X^2 (p-value)
**Age**	Yes	No		Yes	No	
0 - 6 months	43 (56.6)	29 (65.9)	1.69 (0.430)	196 (90.7)	129 (91.5)	0.07 (0.964)
7 - 12 months	20 (26.3)	11 (25.0)		17 (7.9)	10 (7.1)	
13 months and above	13 (17.1)	4 (9.1)		3 (1.4)	2 (1.4)	
**Sex**						
Male	35 (46.1)	22 (50.0)	0.17 (0.676)	122 (56.5)	82 (58.2)	0.10 (0.755)
Female	41 (53.9)	22 (50.0)		94 (43.5)	59 (41.8)	
**Breastfeeding**						
Yes	36 (47.4)	18 (40.9)	0.47 (0.493)	150 (69.4)	105 (74.5)	1.06 (0.304)
No	40 (53.6)	26 (59.1)		66 (30.6)	36 (25.5)	
**Location**						
Alakia	3 (3.9)	3 (6.8)	0.79 (0.939)	7 (3.2)	7 (5.0)	1.57 (0.814)
Ejioku	1 (1.3)	1 (2.3)		5 (2.3)	5 (3.5)	
Eleweodo	15 (19.7)	9 (20.5)		6 (2.8)	4 (2.8)	
Lalupon	33 (43.4)	19 (43.2)		156 (72.2)	102 (72.3)	
Monatan	24 (31.6)	12 (27.3)		42 (19.5)	23 (16.3)	
**BMI**						
Underweight	59 (77.6)	28 (63.6)	2.74 (0.098)	165 (76.7)	107 (76.4)	0.01 (0.945)
Not Underweight	17 (22.4)	16 (36.4)		50 (23.3)	33 (23.6)	
**Household water**						
Sanitary water	0 (0)	1 (2.3)	1.74 (0.187)	1 (0.5)	0 (0.0)	0.66 (0.418)
Unsanitary water	76 (100)	43 (97.7)		215 (99.5)	141 (100.0)	
**Education**						
less than basic education	13 (17.1)	12 (27.3)	1.71 (0.192)	56 (25.9)	43 (30.5)	0.89 (0.346)
Basic education	63 (82.9)	32 (72.7)		160 (74.1)	98 (69.5)	

Supplementary Table 2: Social Demographic Characteristics of Children with EAEC pathotypes

Variable	Case			Control		
	EAEC		X^2 (p-value)	EAEC		X^2 (p-value)
**Age**	Yes	No		Yes	No	
0 - 6 months	41 (56.9)	31 (64.6)	0.70 (0.705)	185 (90.2)	140 (92.1)	0.39 (0.825)
7 - 12 months	20 (27.8)	11 (22.9)		17 (8.3)	10 (6.6)	
13 months and above	11 (15.3)	6 (12.5)		3 (1.5)	2 (1.3)	
**Sex**						
Male	33 (45.8)	24 (50.0)	0.20 (0.654)	118 (57.6)	86 (56.6)	0.03 (0.853)
Female	39 (54.2)	24 (50.0)		87 (42.4)	66 (43.4)	
**Breastfeeding**						
Yes	34 (47.2)	20 (41.7)	0.36 (0.549)	142 (69.3)	113 (74.3)	1.10 (0.294)
No	38 (52.8)	28 (58.3)		63 (30.7)	39 (25.7)	
**Location**						
Alakia	3 (4.2)	3 (6.3)	0.42 (0.981)	7 (3.4)	7 (4.6)	1.77 (0.778)
Ejioku	1 (1.4)	1 (2.1)		4 (2.0)	6 (3.9)	
Eleweodo	15 (20.8)	9 (18.8)		6 (2.9)	4 (2.6)	
Lalupon	31 (43.1)	21 (43.8)		149 (72.7)	109 (71.7)	
Monatan	22 (30.6)	14 (29.2)		39 (19.0)	26 (17.1)	
**BMI**						
Underweight	56 (77.8)	31 (64.6)	0.70 (0.705)	157 (77.0)	115 (76.2)	0.39 (0.825)
Not Underweight	16 (22.2)	17 (35.4)		47 (23.0)	36 (23.8)	
**Household water**						
Sanitary water	0 (0)	1 (2.1)	1.51 (0.219)	1 (0.5)	0 (0.0)	0.74 (0.389)
Unsanitary water	72 (100)	47 (97.9)		204 (99.5)	152 (100.0)	
**Education**						
less than basic education	11 (15.3)	14 (29.2)	3.37 (0.066)	54 (26.3)	45 (29.6)	0.46 (0.496)
Basic education	61 (84.7)	34 (70.8)		151 (73.7)	107 (70.4)	

Supplementary Table 3: Social Demographic Characteristics of Children with ETEC pathotypes

Variable	Case			Control		
	ETEC		X^2 (p-value)	ETEC		X^2 (p-value)
**Age**	Yes	No		Yes	No	
0 - 6 months	3 (37.5)	69 (61.6)	9.08 (**0.011)**	10 (90.9)	315 (91.0)	0.19 (0.908)
7 - 12 months	1 (12.5)	30 (26.8)		1 (9.1)	26 (7.5)	
13 months and above	4 (50.0)	13 (11.6)		0 (0)	5 (1.4)	
**Sex**						
Male	2 (25.0)	55 (49.1)	1.74 (0.187)	5 (45.5)	199 (97.5)	0.63 (0.426)
Female	6 (75.0)	57 (50.9)		6 (54.5)	147 (42.5)	
**Breastfeeding**						
Yes	4 (50.0)	50 (44.6)	0.09 (0.769)	9 (81.8)	246 (71.1)	0.60 (0.438)
No	4 (50.0)	62 (55.4)		2 (18.2)	100 (28.9)	
**Location**						
Alakia	0 (0.0)	6 (5.4)	2.02 (0.732)	0 (0.0)	14 (4.0)	11.35 (**0.023)**
Ejioku	0 (0.0)	2 (1.8)		2 (18.2)	8 (2.3)	
Eleweodo	1 (12.5)	23 (20.5)		0 (0)	10 (2.9)	
Lalupon	3 (37.5)	49 (43.8)		6 (54.5)	252 (72.8)	
Monatan	4 (50.0)	32 (28.6)		3 (27.3)	62 (17.9)	
**BMI**						
Underweight	7 (87.5)	80 (71.4)	0.97 (0.325)	10 (90.9)	262 (96.3)	1.29 (0.255)
Not Underweight	1 (12.5)	32 (28.6)		1 (9.1)	82 (23.8)	
**Household water**						
Sanitary water	0 (0)	1 (0.9)	0.07 (0.788)	0 (0)	1 (0.3)	0.03 (0.858)
Unsanitary water	8 (100)	111 (99.1)		11 (100)	345 (96.9)	
**Education**						
less than basic education	4 (50.0)	21 (18.8)	4.42 (**0.035)**	2 (18.2)	97 (28.0)	0.52 (0.472)
Basic education	4 (50.0)	91 (81.3)		9 (81.8)	249 (72.0)	

Supplementary Table 4: Social Demographic Characteristics of Children with EPEC pathotypes

Variable	Case			Control		
	EPEC		X^2 (p-value)	EPEC		X^2 (p-value)
**Age**	Yes	No		Yes	No	
0 - 6 months	4 (57.1)	68 (60.2)	1.90 (0.387)	15 (93.8)	310 (90.9)	0.29 (0.867)
7 - 12 months	3 (42.9)	28 (24.8)		1 (6.2)	26 (7.6)	
13 months and above	0 (0.0)	17 (15.0)		0 (0.0)	5 (1.5)	
**Sex**						
Male	4 (57.1)	53 (46.9)	0.28 (0.599)	10 (62.5)	194 (56.9)	0.20 (0.658)
Female	3 (42.9)	60 (53.1)		6 (37.5)	147 (43.1)	
**Breastfeeding**						
Yes	2 (28.6)	52 (46.0)	0.81 (0.368)	10 (62.5)	245 (71.8)	0.80 (0.370)
No	5 (71.4)	61 (54.0)		6 (37.5)	96 (28.2)	
**Location**						
Alakia	0 (0.0)	6 (5.3)	0.949 (0.917)	0 (0.0)	14 (4.1)	3.69 (0.450)
Ejioku	0 (0.0)	2 (1.8)		0 (0.0)	10 (2.9)	
Eleweodo	1 (14.3)	23 (20.4)		1 (6.3)	9 (2.6)	
Lalupon	4 (57.1)	48 (42.5)		14 (87.5)	244 (71.6)	
Monatan	2 (28.6)	34 (30.1)		1 (6.3)	64 (18.8)	
**BMI**						
Underweight	7 (100)	80 (70.8)	2.82 (0.093)	10 (62.5)	262 (77.3)	1.87 (0.172)
Not Underweight	0 (0.0)	33 (29.2)		6 (37.5)	77 (22.7)	
**Household water**						
Sanitary water	0 (0.0)	1 (0.9)	0.06 (0.803)	0 (0.0)	1 (0.3)	0.05 (0.828)
Unsanitary water	7 (100)	112 (99.1)		16 (100)	340 (99.7)	
**Education**						
less than basic education	3 (42.9)	22 (19.5)	2.19 (0.139)	4 (25.0)	95 (27.9)	0.06 (0.803)
Basic education	4 (57.1)	91 (80.5)		12 (75.0)	246 (72.1)	

Supplementary Table 5: Logistic Regression of selected demographics

	ETEC (Case)		ETEC (Control)	
**Variable**	**OR (95% CI)**	**P value**	**OR (95% CI)**	**P value**
**Age**				
0 - 6 months (ref)			ND	
7 - 12 months	0.77(0.077-7.673)	0.821	ND	
13 months and above	7.08(1.415-35.404)	**0.017**	ND	
**Location**				
Alakia	ND		0.00(0.000-0.000)	0.999
Ejioku	ND		5.17(0.746-35.766)	0.096
Eleweodo	ND		0.00(0.000-0.000)	0.999
Lalupon	ND		0.49(0.120-2.023)	0.325
Monatan (ref)	ND			
**Education**				
less than basic education	4.33(1.001-18.750)	**0.050**	ND	
Basic education (ref)			ND	

*Empty Cells are either references or cells with zero values and ND means not determined.
